# Supplementary material for: Community-identified priorities for parenting and parental mental health in coastal Ghana: Formative insights to shape a multi-component intervention
Source: PLOS Glob Public Health. 2026 Feb 3;6(2):e0005915. doi: 10.1371/journal.pgph.0005915 (PMC12867211; doi:10.1371/journal.pgph.0005915)
Supplement: Attachment — Submitted filename: Response to reviewers_R1.docx [file pgph.0005915.s004.docx]

Monday, 6^th^ October 2025

PLoS Global Public Health

Editorial Office

Dear Dr. Acharya,

Thank you for the opportunity to revise our manuscript PGPH-D-25-01851 entitled “Community-identified priorities for parenting and parental mental health in coastal Ghana: Formative insights to shape a multi-component intervention” (former title “Formative research to inform the design of a multi-component parenting and parental mental health intervention in Ghana”). We are grateful for the reviewers’ insightful comments, which have significantly strengthened our paper. Please see attached for our point-by-point responses to each of the comments. We have revised the manuscript as requested and indicated revisions in tracked changes in the attached manuscript.

Specifically, we have strengthened the introduction with comparison to global existing data, provided clarity on data collection processes in the methods, provided a clear overview of themes and subthemes in the results, simplified complex sentences in the discussion, and revised the conclusion to better highlight next steps. We have also addressed all of the journal requirements comments. In addition to the revised manuscript with tracked changes, we have attached a clean manuscript without tracked changes. We have also made editorial changes to improve the readability of the manuscript (e.g., using the more specific term ‘nonparental caregiver’ instead of ‘other caregiver’).

We hope that this revised version meets the publication standards of *PLoS Global Public Health* and look forward to hearing from you.

Sincerely,

Marilyn N. Ahun, PhD

Assistant Professor

Department of Medicine

Faculty of Medicine and Health Sciences

McGill University

**Journal requirements**

1. Please include a complete copy of PLOS’ questionnaire on inclusivity in global research in your revised manuscript. Our policy for research in this area aims to improve transparency in the reporting of research performed outside of researchers’ own country or community. The policy applies to researchers who have travelled to a different country to conduct research, research with Indigenous populations or their lands, and research on cultural artefacts. The questionnaire can also be requested at the journal’s discretion for any other submissions, even if these conditions are not met. Please find more information on the policy and a link to download a blank copy of the questionnaire here: <https://journals.plos.org/globalpublichealth/s/best-practices-in-research-reporting>. Please upload a completed version of your questionnaire as Supporting Information when you resubmit your manuscript.

- **Response**: We have completed the questionnaire and uploaded it as supporting information.

2. Please ensure that your Ethics Statement is available in its entirety at the beginning of your Methods section, under a subheading 'Ethics Statement'.

- **Response**: We have moved the ethics statement to the beginning of the Methods section and updated the subheading to read ‘Ethics Statement’.

3. We have noticed that you have uploaded Supporting Information files, but you have not included a list of legends. Please add a full list of legends for your Supporting Information files after the references list.

- **Response**: We have added a full list of legends for our Supporting Information files after the references list.

4. For studies involving third-party data, we encourage authors to share any data specific to their analyses that they can legally distribute. PLOS recognizes, however, that authors may be using third-party data they do not have the rights to share. When third-party data cannot be publicly shared, authors must provide all information necessary for interested researchers to apply to gain access to the data. (<https://journals.plos.org/plosone/s/data-availability#loc-acceptable-data-access-restrictions>). For any third-party data that the authors cannot legally distribute, they should include the following information in their Data Availability Statement upon submission: (1) A description of the data set and the third-party source; (2) If applicable, verification of permission to use the data set; (3) Confirmation of whether the authors received any special privileges in accessing the data that other researchers would not have; (4) All necessary contact information others would need to apply to gain access to the data.

- **Response**: This study does not involve the use of third-party data. All data were collected expressly for the purpose of this study by authors and their research teams.

5. If the reviewer comments include a recommendation to cite specific previously published works, please review and evaluate these publications to determine whether they are relevant and should be cited. There is no requirement to cite these works unless the editor has indicated otherwise.

- **Response**: Thank you for this clarification. We have only included publications that are relevant to our study and merit citation in this manuscript.

6. Please review your reference list to ensure that it is complete and correct. If you have cited papers that have been retracted, please include the rationale for doing so in the manuscript text, or remove these references and replace them with relevant current references. Any changes to the reference list should be mentioned in the rebuttal letter that accompanies your revised manuscript. If you need to cite a retracted article, indicate the article’s retracted status in the References list and also include a citation and full reference for the retraction notice.

- **Response**: We have reviewed our reference list and confirm that it is complete and correct. We have also indicated all changes to the reference list in tracked changes and mentioned them in the rebuttal letter. We have not cited any papers that have been retracted.

**Reviewer #1**

This is a detailed and well-written manuscript. It explores the link between parenting and parental mental health. Given the post-COVID socio-economic turbulence which has significantly affected families and caregiving in LMICs, this study is timely, important and offers meaningful insights. The inclusion of diverse caregivers is positive.

**Response**: Thank you for your time and insightful comments. Please see below for our point-by-point responses. Page numbers refer to the manuscript version with tracked changes.

Areas to note:

1. Title alignment - consider refining the title to better reflect the findings and contributions to make it more outcome-driven and results-oriented.

- **Response**: We have refined the title as suggested. The study title is now “*Community-identified priorities for parenting and parental mental health in coastal Ghana: Formative insights to shape a multi-component intervention*”

2. Key-words - Look at the keywords again, the manuscript contains more powerful keywords. eg "mental health" consider "parental mental health" focusing on parents' psychological well-being covered in the manuscript.

- **Response**: We have revised the keywords as suggested, including the addition of a keyword on global health.
- *Page 2, lines 58-59*: “Keywords: parenting, parental mental health, multi-component intervention, qualitative research, global health, Ghana”

3. Introduction-This section needs stronger comparison with existing global data.

- **Response**: We have revised the introduction to provide more examples of global research and how they connect to the present study.
- *Page 3, lines 78-106*: “Although multi-component interventions promise improvements in both parenting and parental mental health, reviews of interventions from both high- and low-resource contexts reveal that most fall short, often due to an overemphasis on parenting strategies with limited attention to mental health (Al Sager et al., 2024; Goodman & Garber, 2017; Pitchik et al., 2020). This is evident in the findings of a meta-analysis on the impact of multi-component interventions on caregiver outcomes, which found no significant impact on reducing caregiver depressive symptoms (Al Sager et al., 2024). Furthermore, only a small handful of interventions assessed parenting practices, preventing a meta-analysis of intervention impact on this outcome. A closer look at the content of these multi-component interventions reveals that those with only one or two sessions focused on parental mental health had limited impacts on improving both caregiver and child mental health outcomes (Barnhart et al., 2021; Rockers et al., 2016). Existing approaches that simply ‘add on’ one or two mental health sessions to parenting curricula (or vice versa) represent tokenistic integration rather than systematic design informed by prior evidence and caregiver needs. This superficial integration likely explains their limited effectiveness.

Another important limitation of prior interventions is that few are grounded in formative research that explores how interventions can be tailored to local realities (Ahun et al., 2024a). Formative research consists of gathering information on relevant behaviours in a given context, including if and how those behaviours are practiced, barriers and facilitators to engaging in those behaviours, and the existence and implementation of services targeting the behaviours (Craig et al., 2008). Such work is vital in the development of any intervention, and failure to engage in this process of contextual adaptation risks cultural misalignment and reduces effectiveness. For example, a US-based multi-component intervention trialled in northern Ghana failed to improve maternal depression or children’s socio-emotional development, likely due to its lack of contextual relevance (Baumgartner et al., 2021). Specifically, the intervention only targeted mothers, presuming that they were children’s sole caregivers. In collectivistic contexts such as Ghana, omitting fathers and nonparental caregivers overlooks key actors in children’s daily environment and their role in influencing child development (Cuartas et al., 2020; Ghana Statistical Service, 2018).”

- *Added references:*

Barnhart, D. A., Farrar, J., Murray, S. M., Brennan, R. T., Antonaccio, C. M., Sezibera,

V., ... & Betancourt, T. S. (2020). Lay-worker delivered home visiting promotes

early childhood development and reduces violence in Rwanda: a randomized

pilot. *Journal of Child and Family Studies, 29*(7), 1804-1817. DOI:

<https://doi.org/10.1007/s10826-020-01709-1>

Craig, P., Dieppe, P., Macintyre, S., Michie, S., Nazareth, I., & Petticrew, M. (2008).

Developing and evaluating complex interventions: the new Medical Research

Council guidance. *BMJ*, 337. DOI: <https://doi.org/10.1136/bmj.a1655>

Rockers, P. C., Zanolini, A., Banda, B., Chipili, M. M., Hughes, R. C., Hamer, D. H., &

Fink, G.(2018). Two-year impact of community-based health screening and

parenting groups on child development in Zambia: Follow-up to a cluster

randomized controlled trial. *PLoS Medicine, 15*(4), e1002555. DOI:

<https://doi.org/10.1371/journal.pmed.1002555>

4. Methods - COREQ (provide reference).

- **Response**: We have added the reference for the COREQ checklist (page 4, lines 137-138) to the manuscript: Tong, A., Sainsbury, P., & Craig, J. (2007). Consolidated criteria for reporting qualitative research (COREQ): A 32-item checklist for interviews and focus groups. *International Journal for Quality in Health Care, 19*(6).

5. Sampling -i. Clarify whether mobilizers followed a standardized recruitment guide to avoid biases. ii. Clarify how you ensured diversity and equal representation of voices. iii. Consider a brief explanation of why 6-60months range was chosen.

- **Response**: To minimize recruitment biases, all community mobilizers were trained with and followed a standardized recruitment guide that outlined the study’s eligibility criteria, communication protocols, and procedures for approaching potential participants. The guide provided clear instructions on how to introduce the study, obtain informed consent, and respond to frequently asked questions, thereby ensuring consistency across mobilizers and study sites. In addition, deliberate steps were taken to ensure diversity and equal representation of voices. Recruitment targeted parents and caregivers across varied socio-demographic backgrounds, including differences in gender, age, marital status, educational attainment, and household socioeconomic status. This approach was reinforced through periodic debrief meetings with mobilizers to review recruitment patterns and ensure that no subgroup was under-represented. Thus, the final sample reflected a broad cross-section of caregiver perspectives within the study communities. We have briefly clarified these procedures in the methods section and also included an explanation of why we recruited caregivers of children between the ages of 6-60 months.
- *Page 5, lines 152-160*: “A purposive sampling strategy was employed to ensure representation of diverse caregiving perspectives across various sociodemographic characteristics (e.g., gender, age, educational attainment, socioeconomic status, etc.). We engaged three community mobilizers, identified by local assembly and health officials, to recruit participants based on their familiarity with household caregiving dynamics in study areas. Mobilizers were trained with and followed a standardized recruitment guide that outlined the study’s eligibility criteria, communication protocols, and procedures for approaching potential participants (Appiah et al., 2025). The project coordinator (DDK) met periodically with mobilizers throughout recruitment to ensure adherence to the guide and diversity of recruited participants.”
- *Page 5, lines 166-168*: “We recruited caregivers of children 6-60 months because the first five years of life are a critical period for brain development, where children are particularly sensitive to their environment (Black et al., 2017).”
- *Added references*:

Appiah, R., Weobong, B., & Raviola, G. (2025). Bridging ethics and culture: a co-created,

culturally sensitive informed consent framework for research in Bimoba and Mamprusi

communities in Ghana. *Ethics & Behavior*, 1-18. DOI:

<https://doi.org/10.1080/10508422.2025.2512069>

6. The discussion session is wordy. Shorten complex sentences to improve readability.
   Overall, it is a strong piece of work, these minor revisions will enhance rigorousness.

- **Response**: Thank you for your helpful feedback. We have revised the discussion as suggested to improve readability. Due to space considerations, we only include a few examples of these changes here.
- *Page 18, lines 648-651*: “We found that financial stress was a key driver of parental distress and limited engagement with children, particularly among fathers. This suggests that interventions that incorporate economic strengthening, such as cash transfers, may enhance both participation and impact.”
- *Page 19, lines 677-683*: “Some fathers in our study recognized the importance of shared caregiving and voiced frustration at community stigma around men performing domestic roles. This suggests that some community members may be open to engaging in norm-shifting conversations. Evidence shows that interventions with gender-transformative approaches lead to more equitable division of responsibilities and paternal engagement (Doyle et al., 2018; Gage et al., 2022; Jeong et al., 2016, 2023). This is crucial for improving ECD.”

**Reviewer #2**

**Overall Assessment**

This manuscript addresses an important public health issue by exploring the socio-cultural and structural factors influencing parenting and parental mental health in southern Ghana, with the aim of informing the design of a multi-component intervention. The introduction is well-structured, providing a strong rationale that situates the problem within global, regional, and local contexts, and highlights critical gaps in culturally adapted interventions. The study objectives are clearly articulated and directly aligned with these gaps.

The methods demonstrate rigor and transparency, with a well-described qualitative design, detailed sampling strategies, comprehensive data collection procedures, and adherence to ethical standards. The inclusion of COREQ guidelines and reflexivity considerations further strengthens the credibility of the approach. The results section is rich in detail, presenting nuanced findings supported by illustrative participant quotes and organized into clear thematic categories that align with the research objectives.

The discussion offers a thoughtful interpretation of findings, effectively linking them to broader literature and emphasizing their implications for intervention design, particularly the need for gender-responsive and community-based strategies. The conclusion appropriately synthesizes key insights and underscores their relevance for policy and practice.

This study is timely, methodologically sound, and of practical significance for designing culturally appropriate parenting and mental health programs in similar contexts. However, some refinements would further enhance its clarity and scientific contribution.

**Strengths**

The manuscript demonstrates several notable strengths. The introduction provides a strong rationale by situating the issue of suboptimal early childhood development (ECD) within both global and national contexts. It effectively highlights the connections between caregiver mental health, parenting practices, and child outcomes, and addresses the common limitation of poor contextual adaptation in multi-component interventions. The inclusion of an example from northern Ghana (Baumgartner et al., 2021) adds depth to the argument for context-specific solutions. The purpose of the study is clearly articulated, and the selection of a descriptive phenomenological design is appropriate for exploring caregivers’ lived experiences. Sampling strategies, engagement with community mobilizers, and well-defined inclusion criteria further enhance contextual relevance. Additionally, the detailed description of interview guide development, piloting, interviewer training, and quality control measures strengthens methodological rigor and credibility.

The analytic process is presented with commendable transparency. Thematic analysis procedures, including codebook development, iterative coding, and consensus-building through regular team meetings, reflect good qualitative research practice. The authors also acknowledge researcher positionality and cultural familiarity, and the inclusion of analysts with lived experience is a noteworthy strength. Ethical rigor is evident through IRB approvals, informed consent processes, and participant appreciation tokens. The results are presented in a rich and organized manner, supported by verbatim quotes and diverse perspectives from mothers, fathers, and community members. Themes are clearly structured and aligned with the study objectives. The discussion effectively integrates findings with existing literature and provides actionable recommendations, including the integration of economic strengthening, gender-transformative approaches, and community-based mental health support. Practical suggestions, such as partnering with faith-based institutions and introducing low-intensity interventions, enhance applied relevance.

Overall, the manuscript offers important insights into how cultural norms, gender dynamics, and economic realities shape caregiving roles, while highlighting emerging changes such as evolving paternal involvement. However, several areas could be strengthened to improve clarity, engagement, and contextual relevance.

**Response**: Thank you for taking the time to conduct a thorough review of our manuscript and for your detailed and insightful comments. Please see below for our point-by-point responses to your suggestions for improvement. Page numbers refer to the manuscript version with tracked changes.

**Areas for Improvement and suggestions for improvement:**

1. References to Ghana Health Service reports from 2012 and 2015 may appear outdated. Please update these figures using more recent sources such as UNICEF, WHO, or DHS reports, or explicitly acknowledge the lack of updated national estimates.

- **Response**: We have provided more recent Ghanaian sources for these data. Please note that although an updated census was conducted in 2021 (and is now cited in the manuscript), the regional reports have not yet been made publicly available, and we therefore maintain the citation from the previous census report.
- *Page 3, lines 64-66*: “In Ghana, a lower-middle-income country in West Africa, this figure ranges from 40–59%, underscoring the urgency for early interventions (Ghana Statistical Service, 2018).”
- *Page 4, lines 141-143*: “The study took place in Kokrobite, Tuba, and Bortianor, fishing communities in Ghana’s Ga South district, where gendered labour roles and socio-economic constraints shape family life (Ghana Statistical Service, 2013, 2022; Ghana Statistical Service and ICF, 2024).”
- *Added references:*

Ghana Statistical Service. (2013). *2010 Population and Housing Census: Regional*

*Analytical Report for the Greater Accra Region*. Ghana Statistical Service.

Ghana Statistical Service. (2018). *Multiple Indicator Cluster Survey (MICS2017/18),*

*survey findings report.* Ghana Statistical Service.

Ghana Statistical Service. (2022). *Ghana 2021 Population and Housing Census: Volume*

*3. General Report Highlights*. Ghana Statistical Service.

Ghana Statistical Service (GSS) and ICF. (2024). *Ghana Demographic and Health*

*Survey 2022.* Accra, Ghana, and Rockville, Maryland, USA: GSS and ICF.

2. The explanation of why interventions often fail (i.e., cultural misalignment) feels generic. Adding one or two sentences on specific sociocultural factors in Ghana (such as norms around parenting or mental health stigma) would provide stronger context.

- **Response**: We have added a few sentences on the specific sociocultural factors in Ghana that may explain why interventions developed for other contexts fail.
- *Page 3, lines 100-106*: “For example, a US-based multi-component intervention trialled in northern Ghana failed to improve maternal depression or children’s socio-emotional development, likely due to its lack of contextual relevance (Baumgartner et al., 2021). Specifically, the intervention only targeted mothers, presuming that they were children’s sole caregivers. In collectivistic contexts such as Ghana, omitting fathers and nonparental caregivers overlooks key actors in children’s daily environment and their role in influencing child development (Cuartas et al., 2020; Ghana Statistical Service, 2018).”

3. Several sentences contain multiple clustered citations, particularly in lines 66–67 and 71–73. Consider consolidating these by prioritizing the most relevant references for improved readability.

- **Response**: We have done our best to consolidate sentences with multiple clustered citations where possible (e.g., removal of Grantham-McGregor et al., 2007 and Walker et al., 2011 on page 3, line 69). For sentences where multiple citations are necessary to the topics discussed in the sentence, we have left the citations as is. For example, on page 3, lines 74-76, we cite three reviews that provide differing and complementary pieces of evidence on how standalone parenting or mental health interventions result in sustained, holistic improvements in caregiver and child well-being: Jeong et al., 2018 refers to parenting interventions (for mothers and fathers), Goodman et al., 2018 to mental health interventions for mothers, and Rominov et al., 2016 to mental health interventions for fathers.

4. The link between identified gaps and the choice of Responsive Parenting and Inspired Life programs is abrupt. Please provide a brief rationale for selecting these programs (e.g., prior evidence of effectiveness or adaptability to similar contexts).

- **Response**: We agree with the reviewer that the introduction of these interventions was abrupt. Upon further reflection, we have decided to remove references to these interventions as they do not inform the study’s rationale or the discussion of our findings. We have instead provided more details on the study’s aims.
- *Page 4, lines 108-114*: “In light of these gaps, this study aims to generate formative evidence on parenting and parental mental health in Ghana to guide the development of a contextually-relevant multi-component intervention that addresses families’ needs. Specifically, we aim to (1) understand how parents and nonparental caregivers care for their young children and support their own mental health, (2) identify the factors that facilitate or hinder their engagement in these behaviours, and (3) examine community services to support parents and nonparental caregivers. We use the term “caregivers” to refer to both parents and nonparental caregivers.”

5. The methods section does not report the total number of IDIs, FGDs, and participants. This omission makes it difficult to assess whether data saturation was achieved. Please include these figures and describe the sampling rationale (e.g., data saturation or other criteria).

- **Response**: The total number of IDIs, FGDs, and participants was previously reported in the results. We have now moved this information to the methods and described the sampling rationale.
- *Page 5, lines 171-176*: “Out of 91 people contacted, 83 agreed to participate. The primary reason for refusal was survey fatigue. The sample size was based on other qualitative studies of a similar nature and adapted during data collection through the monitoring of results to assess whether findings had reached a point of saturation (Ahun et al., 2021, 2024b; Jeong et al., 2023). We conducted 31 IDIs with fathers, mothers, nonparental caregivers, and community members and 6 FGDs with 52 participants across father-only, mother-only, and mixed-parent groups (Table 1).”

6. While Kokrobite, Tuba, and Bortianor are described as fishing communities, the rationale for choosing these sites over others in Ghana is unclear. Consider explaining their relevance to the research questions (e.g., prevalence of mental health issues or representativeness of similar communities).

- **Response**: We have provided a rationale for choosing these sites in the methods. Our decision was based on the representativeness of these sites of other coastal towns and our established relationship with community elders.
- *Page 4, lines 141-149*: “The study took place in Kokrobite, Tuba, and Bortianor, fishing communities in Ghana’s Ga South district, where gendered labour roles and socio-economic constraints shape family life (Ghana Statistical Service, 2013, 2022; Ghana Statistical Service and ICF, 2024).This site was chosen due to its sociodemographic similarities with other coastal towns and the authors’ established relationships with community elders through previous research (Ahun et al., under review; Ghana Statistical Service, 2013). These peri-urban areas report high rates of socioemotional difficulties among children (47%) and significant parental mental health difficulties, with maternal and paternal depression estimated at 35% and 26%, respectively (Ahun et al., under review; Paul et al., in preparation).”
- *Added references*:

Ghana Statistical Service. (2013). *2010 Population and Housing Census: Regional*

*Analytical Report for the Greater Accra Region*. Ghana Statistical Service.

Ghana Statistical Service. (2022). *Ghana 2021 Population and Housing Census: Volume*

*3. General Report Highlights*. Ghana Statistical Service.

Ghana Statistical Service (GSS) and ICF. (2024). *Ghana Demographic and Health*

*Survey 2022.* Accra, Ghana, and Rockville, Maryland, USA: GSS and ICF.

Paul, I., Pai, N.P., Appiah, R., & Ahun, M.N. (in preparation). Prevalence and correlates

of maternal and paternal mental health in Ghana

7. The description of translation and Ga-Dangme adaptation lacks detail on how accuracy was maintained during transcription and analysis. Please clarify whether methods such as back-translation or bilingual checks were employed.

- **Response**: The project coordinator, who is a native speaker of Ga-Dangme, did bilingual checks of all the transcripts by comparing the transcripts with the audio files. This ensured that the transcripts were free of errors and met the research standards. We have clarified this in the methods.
- *Page 6, lines 202-204*: “Transcripts and field notes were uploaded at the end of each day of data collection, translated, and double-checked by the project coordinator, a native Ga-Dangme speaker, to ensure accuracy and minimize errors.”

8. Information on the average duration of interviews/FGDs and whether data saturation guided data collection is missing. Including these details would improve transparency.

- **Response**: This information was previously reported in the results. We have now moved it to the methods section.
- *Page 5, lines 171-177*: “Out of 91 people contacted, 83 agreed to participate. The primary reason for refusal was survey fatigue. The sample size was based on other qualitative studies of a similar nature and adapted during data collection through the monitoring of results to assess whether findings had reached a point of saturation (Ahun et al., 2021, 2024b; Jeong et al., 2023). We conducted 31 IDIs with fathers, mothers, nonparental caregivers, and community members and 6 FGDs with 52 participants across father-only, mother-only, and mixed-parent groups (Table 1). On average, IDIs lasted 48 minutes and FGDs lasted 1 hour 51 minutes.”

9. Major themes are labeled, but subthemes are inconsistently formatted and not easy to scan. Please use clear subheadings for all subthemes to enhance structure and readability. Each major theme should explicitly relate back to the research objectives stated in the introduction. Consider adding a brief statement at the start of each section to clarify this linkage. Currently, tables only present demographic data. Adding a summary table of themes and subthemes with brief descriptions and representative quotes would help readers quickly grasp the findings.

- **Response**: We have revised the results section to better label major themes and consistently format subthemes and subheadings. We have added an overview paragraph to explain the themes and subthemes, as well as how they relate back to the research objectives stated in the linkage. We have also added a table to help readers quickly grasp the findings.
- *Pages 8-9, lines 265-275*: “Findings are organized into four broad themes (and their sub-themes) in line with our specific aims: (1) overall parenting experiences, (2) positive parenting behaviours, (3) parental mental health, and (4) existing community services. Sub-themes on current practices within themes 2 (positive parenting behaviours) and 3 (parental mental health) address our first specific aim by describing how caregivers care for their young children and support their own mental health. Within each of these themes, participants also discuss the specific barriers and facilitators caregivers face when engaging in each of these behaviours, shedding light on specific aim 2. Additionally, theme 4 allows an examination of community services to support caregivers, as well as caregivers’ experiences accessing them (specific aim 3). The first theme on overall parenting experiences provides helpful context within which to interpret all other themes. These themes and sub-themes are summarized in Table 3.

**Table 3.** Major themes and sub-themes

| **Themes** | **Sub-themes** | **Example quotes** |
| --- | --- | --- |
| Parenting experiences | | *“A father can take care of the child, but you can’t be compared to the mother, do you get me. That’s why they say sweet mother [referring to a well-known song], do you get me, uh huh.”* *(Father-IDI-#1* |
| Positive parenting behaviours | Practices | *“I give them house training. And because I didn’t go to school the house training my* *father taught me is what once a while I remember...like, uh huh, for instance like advising* *you not to join any bad company, joining gang in school. ” (Father-IDI-#6).* |
|  | Facilitators | *“It’s like the church does that in terms of programs and then when people come to church on Sunday, we try to educate them about most of the social challenges...” (Healthcare worker-IDI-#8)* |
|  | Barriers | *“I do remember, my daughter, my first born she wanted to tell us her aspiration but* *because the father doesn’t stay at home she can’t tell the father. So, she wrote it on a* *paper and posted in the room, when you enter the room, you will see it posted there, she* *wrote and pasted it on the wall.” (Mixed FGD-#13).* |
| Parental mental health | Practices | *“I try not to rely on anybody. I pray to God, sometimes he uses people to help me. That sometimes makes the sadness go away and I’m happy.” (Aunt-IDI-*#16*)* |
|  | Facilitators | *“...But if I work and earn something, I feel happy that whenever any child of mine will face any problem, maybe in terms of an illness or the likes, I have something on me and can solve it for them.” (Father-IDI-#18* |
|  | Barriers | *“There are a lot of needs, that I can be up from morning, but I do not have money to use* *to take care of the family, then it becomes a major problem for me. It becomes a thinking* *problem for me. Then if for twenty-four hours, I do not get any help from anywhere, then* *this problem will be on my mind.” (Father-IDI-#18)* |
| Existing community services | Experiences | *“At times nurses are mobilized to come round the community to talk to us and educate us on how to take care of our children to avoid contracting diseases. There are also two* *polyclinics here where we go to whenever we feel sick.” (Mother-FGD-#27).* |
|  | Interest in additional programs | *“Yes, it depends on how you send the message, who gives the message and where you give the message. Always involve community leaders.” (Healthcare worker-IDI-#8)* |

10. Some sections remain largely descriptive. For example, the discussion on gendered parenting roles lacks reflection on their implications for mental health and parenting quality. Adding short interpretive statements would strengthen analytical depth.

- **Response**: We have added short interpretive statements to the discussion to strengthen the analysis. Due to space considerations, we only include a few examples of these changes here.
- *Page 19, lines 661-667*: “Furthermore, cash transfer interventions across sub-Saharan Africa have reported a decrease in maternal stress and improvements in economic well-being (Ngamasana & Moxie, 2024; Wolf et al., 2024). These findings suggest that addressing financial strain not only supports material conditions but also alleviates psychological distress and promotes positive parent-child relationships.”
- *Page 19, lines 669-674*: “Paternal disengagement also emerged as a significant barrier to positive parenting and maternal well-being. According to participants, this disengagement was driven by time constraints and entrenched gender norms, which left mothers with disproportionate household chores that increased maternal stress. This stress sometimes resulted in displaced frustration towards their children, highlighting how gendered roles can negatively affect both mental health and parenting practices.”

11. The results highlight diverse views but do not explicitly mention minority or contradictory perspectives (e.g., fathers who fully share domestic responsibilities). Including and interpreting these would enrich the analysis.

- **Response**: We have revised the results to better present minority and contradictory perspectives. Due to space considerations, we only include a few examples of these changes here.
- *Page 11, lines 316-319*: “Some caregivers explained that they had not strictly divided their roles but rather believed in supporting one another and sharing tasks based on who is available. Indeed, although less commonly reported, a few fathers expressed gratitude and appreciation for their partners for contributing financially.”
- *Page 13, lines 399-403*: “Another barrier to engaging in positive parenting practices was the lack of paternal involvement.. Although a small number of fathers reported engaging in caregiving more equally, mothers, other female caregivers, and respected community members emphasized that this was not the norm.”

12. The discussion should clearly restate how key themes answer the research questions outlined in the introduction. A short mapping section early in the discussion would improve coherence.

- **Response**: We have revised the first section of the discussion to improve coherence by clearly restating our research objectives and how key themes address them.
- *Page 18, lines 626-636*: “This formative study sheds light on how caregivers in southern Ghana care for young children and their own mental health by providing new insights into how socio-economic and cultural conditions shape parenting and parental mental health. Participants provided rich descriptions of how caregiver engage in parenting practices and support their mental health, the barriers and facilitate they face in doing so, and their experiences of existing community services to support parenting and parental mental health. Findings reveal that financial insecurity, rigid gender norms, and the absence of accessible mental health services undermine both caregiver well-being and child development. Our results highlight the importance of addressing structural and relational challenges to ensure that multi-component parenting and mental health interventions are contextually relevant, gender-responsive, and address local needs.”

13. While some limitations (livelihood context, two-parent households) are mentioned, others are missing. Please discuss potential issues such as social desirability bias, limited generalizability due to purposive sampling, and possible underreporting of sensitive issues (e.g., intimate partner violence).

- **Response**: We acknowledge that these limitations are relevant to our study and have included them in the discussion.
- *Page 20, lines 713-728*: “This study provides novel data on caregivers’ and community members’ perspectives on how caregivers care for their young children and their own mental health in a Ghanaian context. It also sheds light on the barriers and facilitators that caregivers face when engaging in these behaviours. A key strength of this study lies in its inclusion of diverse caregiving voices, from mothers, fathers, nonparental caregivers, and community members, allowing for a multidimensional understanding of parenting and mental health in low-resource, coastal communities. However, the predominance of fishing as a livelihood in the study area may limit applicability to contexts where fathers are more consistently present in the home. Our use of purposive sampling may also limit the generalizability of findings to other populations. Future research could explore whether these patterns persist in urban or agricultural regions. Furthermore, although the combination of IDIs and FGDs provided both individual and collective perspectives on sensitive topics, it is possible that social desirability bias was present and participants may not have felt comfortable discussing some topics (e.g., intimate partner violence), which were thus underreported. Finally, while the sample focused on two-parent households, the inclusion of extended kin caregivers offers some insight into alternative caregiving arrangements common in Ghana.”

14. The conclusion currently repeats portions of the discussion and does not clearly summarize key contributions. Consider making it more concise and focusing on: What this study adds to the literature and the most critical next steps for research and policy.

- **Response**: We have revised the conclusion accordingly.
- *Page 20, lines 731-741*: “This formative qualitative study explored how parents, caregivers, and community members in southern Ghana experience and navigate parenting and mental health difficulties. Findings underscore the need for a contextually-relevant multi-component parenting and parental mental health intervention that combines economic strengthening with gender-transformative and community-based mental health support. Researchers, policymakers, and health providers need to work alongside communities to integrate these findings into the development of policies and interventions to better support Ghanaian families.”

**Conclusion**

This is an important and timely study that offers valuable insights for designing culturally appropriate and gender-responsive parenting interventions in Ghana. The manuscript is strong in many respects, particularly in its rationale, methodological rigor, and practical implications. To make the paper even stronger, I encourage the authors to provide greater methodological detail, refine the structure of the results, and more clearly link findings to theoretical frameworks in the discussion. These refinements will enhance both readability and the scientific contribution of the work.

**Response**: Thank you, once again, for your helpful comments. We have revised the manuscript accordingly to improve its readability and scientific contribution.
