# Supplementary material for: Community-identified priorities for parenting and parental mental health in coastal Ghana: Formative insights to shape a multi-component intervention
Source: PLOS Glob Public Health. 2026 Feb 3;6(2):e0005915. doi: 10.1371/journal.pgph.0005915 (PMC12867211; doi:10.1371/journal.pgph.0005915)
Supplement: Attachment — Submitted filename: Reviewer Comment.pdf [file pgph.0005915.s003.pdf]

Manuscript Review Report

Manuscript Number: PGPH-D-25-01851

Title: “Formative research to inform the design of a multi-component parenting and parental mental health intervention in Ghana”

Overall Assessment

This manuscript addresses an important public health issue by exploring the socio-cultural and structural factors influencing parenting and parental mental health in southern Ghana, with the aim of informing the design of a multi-component intervention. The introduction is well-structured, providing a strong rationale that situates the problem within global, regional, and local contexts, and highlights critical gaps in culturally adapted interventions. The study objectives are clearly articulated and directly aligned with these gaps.

The methods demonstrate rigor and transparency, with a well-described qualitative design, detailed sampling strategies, comprehensive data collection procedures, and adherence to ethical standards. The inclusion of COREQ guidelines and reflexivity considerations further strengthens the credibility of the approach. The results section is rich in detail, presenting nuanced findings supported by illustrative participant quotes and organized into clear thematic categories that align with the research objectives.

The discussion offers a thoughtful interpretation of findings, effectively linking them to broader literature and emphasizing their implications for intervention design, particularly the need for gender-responsive and community-based strategies. The conclusion appropriately synthesizes key insights and underscores their relevance for policy and practice.

This study is timely, methodologically sound, and of practical significance for designing culturally appropriate parenting and mental health programs in similar contexts. However, some refinements would further enhance its clarity and scientific contribution.

Strengths

The manuscript demonstrates several notable strengths. The introduction provides a strong rationale by situating the issue of suboptimal early childhood development (ECD) within both global and national contexts. It effectively highlights the connections between caregiver mental health, parenting practices, and child outcomes, and addresses the common limitation of poor contextual adaptation in multi-component interventions. The inclusion of an example from

northern Ghana (Baumgartner et al., 2021) adds depth to the argument for context-specific solutions. The purpose of the study is clearly articulated, and the selection of a descriptive phenomenological design is appropriate for exploring caregivers' lived experiences. Sampling strategies, engagement with community mobilizers, and well-defined inclusion criteria further enhance contextual relevance. Additionally, the detailed description of interview guide development, piloting, interviewer training, and quality control measures strengthens methodological rigor and credibility.

The analytic process is presented with commendable transparency. Thematic analysis procedures, including codebook development, iterative coding, and consensus-building through regular team meetings, reflect good qualitative research practice. The authors also acknowledge researcher positionality and cultural familiarity, and the inclusion of analysts with lived experience is a noteworthy strength. Ethical rigor is evident through IRB approvals, informed consent processes, and participant appreciation tokens. The results are presented in a rich and organized manner, supported by verbatim quotes and diverse perspectives from mothers, fathers, and community members. Themes are clearly structured and aligned with the study objectives. The discussion effectively integrates findings with existing literature and provides actionable recommendations, including the integration of economic strengthening, gender-transformative approaches, and community-based mental health support. Practical suggestions, such as partnering with faith-based institutions and introducing low-intensity interventions, enhance applied relevance.

Overall, the manuscript offers important insights into how cultural norms, gender dynamics, and economic realities shape caregiving roles, while highlighting emerging changes such as evolving paternal involvement. However, several areas could be strengthened to improve clarity, engagement, and contextual relevance.

Areas for Improvement and suggestions for improvement:

1. References to Ghana Health Service reports from 2012 and 2015 may appear outdated. Please update these figures using more recent sources such as UNICEF, WHO, or DHS reports, or explicitly acknowledge the lack of updated national estimates.
2. The explanation of why interventions often fail (i.e., cultural misalignment) feels generic. Adding one or two sentences on specific sociocultural factors in Ghana (such as norms around parenting or mental health stigma) would provide stronger context.

3. Several sentences contain multiple clustered citations, particularly in lines 66–67 and 71–73. Consider consolidating these by prioritizing the most relevant references for improved readability.
4. The link between identified gaps and the choice of Responsive Parenting and Inspired Life programs is abrupt. Please provide a brief rationale for selecting these programs (e.g., prior evidence of effectiveness or adaptability to similar contexts).
5. The methods section does not report the total number of IDIs, FGDs, and participants. This omission makes it difficult to assess whether data saturation was achieved. Please include these figures and describe the sampling rationale (e.g., data saturation or other criteria).
6. While Kokrobite, Tuba, and Bortianor are described as fishing communities, the rationale for choosing these sites over others in Ghana is unclear. Consider explaining their relevance to the research questions (e.g., prevalence of mental health issues or representativeness of similar communities).
7. The description of translation and Ga-Dangme adaptation lacks detail on how accuracy was maintained during transcription and analysis. Please clarify whether methods such as back-translation or bilingual checks were employed.
8. Information on the average duration of interviews/FGDs and whether data saturation guided data collection is missing. Including these details would improve transparency.
9. Major themes are labeled, but subthemes are inconsistently formatted and not easy to scan. Please use clear subheadings for all subthemes to enhance structure and readability.
10. Each major theme should explicitly relate back to the research objectives stated in the introduction. Consider adding a brief statement at the start of each section to clarify this linkage.
11. Some sections remain largely descriptive. For example, the discussion on gendered parenting roles lacks reflection on their implications for mental health and parenting quality. Adding short interpretive statements would strengthen analytical depth.
12. Currently, tables only present demographic data. Adding a summary table of themes and subthemes with brief descriptions and representative quotes would help readers quickly grasp the findings.
13. The results highlight diverse views but do not explicitly mention minority or contradictory perspectives (e.g., fathers who fully share domestic responsibilities). Including and interpreting these would enrich the analysis.

14. The discussion should clearly restate how key themes answer the research questions outlined in the introduction. A short mapping section early in the discussion would improve coherence.
15. While some limitations (livelihood context, two-parent households) are mentioned, others are missing. Please discuss potential issues such as social desirability bias, limited generalizability due to purposive sampling, and possible underreporting of sensitive issues (e.g., intimate partner violence).
16. The conclusion currently repeats portions of the discussion and does not clearly summarize key contributions. Consider making it more concise and focusing on: What this study adds to the literature and the most critical next steps for research and policy

Conclusion

This is an important and timely study that offers valuable insights for designing culturally appropriate and gender-responsive parenting interventions in Ghana. The manuscript is strong in many respects, particularly in its rationale, methodological rigor, and practical implications. To make the paper even stronger, I encourage the authors to provide greater methodological detail, refine the structure of the results, and more clearly link findings to theoretical frameworks in the discussion. These refinements will enhance both readability and the scientific contribution of the work.
